# Multivariable prediction models for the recovery of and claim closure related to post-collision neck pain and associated disorders

**DOI:** 10.1186/s12998-023-00504-1

**Published:** 2023-08-25

**Authors:** Maja Stupar, Pierre Côté, Linda J. Carroll, Robert J. Brison, Eleanor Boyle, Heather M. Shearer, J. David Cassidy

**Affiliations:** 1 Institute for Disability and Rehabilitation Research, Ontario Tech University, Oshawa, Canada; 2 Division of Graduate Education and Research, Canadian Memorial Chiropractic College, Toronto, Canada; 3 Faculty of Health Sciences, Ontario Tech University, Ontario, Canada; 4 Division of Epidemiology, Dalla Lana School of Public Health, University of Toronto, Toronto, Canada; 5 School of Public Health, University of Alberta, Edmonton, Canada; 6 Kingston General Hospital Research Inst, Kingston, Canada; 7 Department of Emergency Medicine, Faculty of Health Sciences, Queen’s University, Kingston, Canada; 8 Thunderbird Partnership Foundation, Bothwell, ON UK; 9 Holland Bloorview Kids Rehabilitation Hospital, Toronto, Canada

**Keywords:** Neck pain, Whiplash, Clinical prediction model, Rehabilitation, Disability, Health recovery

## Abstract

**Objective:**

Few clinical prediction models are available to clinicians to predict the recovery of patients with post-collision neck pain and associated disorders. We aimed to develop evidence-based clinical prediction models to predict (1) self-reported recovery and (2) insurance claim closure from neck pain and associated disorders (NAD) caused or aggravated by a traffic collision.

**Methods:**

The selection of potential predictors was informed by a systematic review of the literature. We used Cox regression to build models in an incident cohort of Saskatchewan adults (n = 4923). The models were internally validated using bootstrapping and replicated in participants from a randomized controlled trial conducted in Ontario (n = 340). We used C-statistics to describe predictive ability.

**Results:**

Participants from both cohorts (Saskatchewan and Ontario) were similar at baseline. Our prediction model for self-reported recovery included prior traffic-related neck injury claim, expectation of recovery, age, percentage of body in pain, disability, neck pain intensity and headache intensity (C = 0.643; 95% CI 0.634–0.653). The prediction model for claim closure included prior traffic-related neck injury claim, expectation of recovery, age, percentage of body in pain, disability, neck pain intensity, headache intensity and depressive symptoms (C = 0.637; 95% CI 0.629–0.648).

**Conclusions:**

We developed prediction models for the recovery and claim closure of NAD caused or aggravated by a traffic collision. Future research needs to focus on improving the predictive ability of the models.

**Supplementary Information:**

The online version contains supplementary material available at 10.1186/s12998-023-00504-1.

## Background

Predicting prognosis of neck pain and associated disorders (NAD) related to a traffic collision is challenging for clinicians, insurers and policy makers. This is due, in part, to the lack of high-quality evidence about the prognosis of NAD [1]. To date, the best evidence on the prognosis of NAD suggests that half of those with NAD secondary to traffic collisions will improve within three months and recover within six months [2]. Moreover, prognostic factors associated with recovery include post-crash psychological factors such as expectation of poor recovery, pain-related depression, anxiety, fear, frustration or anger and poor coping [2]. Improving the ability of clinicians to predict the outcomes of patients with post-collision neck pain is important because it is estimated that 50% of persons with this condition continue to experience symptoms one-year post-collision [1, 2]. Therefore, our ability to effectively predict the course of NAD is limited.

Clinical prediction models are tools developed to assist clinicians predict clinical outcomes using a combination of patient, clinical and other variables [3]. Using valid clinical prediction models improves prognosis prediction compared to clinical judgement alone [3]. Several models aimed at predicting recovery from NAD secondary to traffic collisions have been developed [4–15]. However, few of these models have been internally [8] or externally validated [5–7, 15, 16] and only one study developed a clinical prediction rule [14]. Therefore, there is a need to develop validated clinical prediction models for NAD secondary to traffic collisions.

Our objective was to develop evidence-based clinical prediction models to predict recovery from NAD secondary to traffic collision. We used the following steps to meet our objective:

1. We developed prediction models in a population-based inception-cohort of Saskatchewan adults injured in traffic collisions.
2. We internally validated the models using bootstrapping methods.
3. We repeated the models in a sample of adults from Ontario enrolled in a randomized controlled trial.

## Methods

We used the TRIPOD Checklist for Prediction Model Development to report our study (Appendix A).

### Selection of potential prognostic Factors/Independent variables

#### Best-evidence synthesis methods to identify prognostic factors

We conducted a systematic review of the literature to identify potential prognostic factors to be included in our development model [2]. In the systematic review, the prognostic factors were classified according to three levels of evidence: (a) Phase I are exploratory studies reporting unadjusted associations; (b) Phase II are exploratory studies reporting associations measured in multivariable models; and (c) Phase III are confirmatory studies that test the independence of associations [1, 17]. Therefore, evidence of association from Phase III studies can be used with more confidence than evidence from Phase I and Phase II studies.

### Summary of the systematic review on prognostic factors

#### Prognostic factors identified from Phase III studies

Based on confirmatory evidence, the following factors were deemed to be independent predictors of delayed recovery or health improvement: prior NAD secondary to a traffic collision, poor expectation of recovery, passive coping and greater levels of depression, anxiety, fear, frustration and/or anger related to the post-collision pain [2].

#### Prognostic factors identified from phase I and II studies

Based on preliminary evidence, older age may be associated with poorer disability recovery. In addition, poor or delayed recovery may be associated with: prior generalized pain, kinesiophobia, cervical radiculopathy, post-crash cold pain threshold, pain intensity and disability, number and severity of symptoms, symptoms of acute stress disorder and post-traumatic stress disorder, and early post-crash onset of depressive symptoms [2].

#### Other prognostic factors

We also considered factors that were not identified as predictors in the systematic review but that clinicians may consider as associated with recovery based on clinical experience (i.e. sociodemographic and clinical factors) [List of variables in Appendix B].

### Development and Internal Validation: the Saskatchewan dataset

We developed the model using data from a population-based, inception cohort of traffic injury from Saskatchewan [18]. Participants with acute NAD were identified by answering “yes” to the question, “Did the accident cause neck or shoulder pain?” [18] We restricted our analysis to participants who provided baseline information within 21 days of the traffic collision. Eligible for the cohort were all Saskatchewan residents who received treatment for a road traffic injury from a regulated health professional (physician, physical therapist, chiropractor or massage therapist) or made an insurance claim for treatment of their injuries to Saskatchewan Government Insurance (SGI) between December 1, 1997 and November 30, 1999. Participants were interviewed approximately 6, 12, 26, 36 and 52 weeks post-injury.

### Replicating: repeating the Models in the Ontario dataset

We replicated our models using data from a randomized clinical trial that investigated the effectiveness of physiotherapy and physician-based care for the management of NAD [19]. The trial was conducted across five primary care clinics in Ontario between February 2008 and April 2011 [19]. Similar to the development cohort, participants were recruited within 21 days of their collision. These participants were also interviewed approximately 6, 12, 26, 36 and 52 weeks post-injury. Blind assessment, sample size and the flow of participants are described elsewhere [19].

### Outcomes

#### Primary outcome: self-reported global recovery

In both studies, recovery was assessed by asking “How well do you feel you are recovering from your injuries?” The response options included: (1) “all better (cured),” (2) “feeling quite a bit improved,” (3) “feeling some improvement,” (4) “feeling no improvement,” (5) “getting a little worse,” or (6) “getting much worse.” [18, 20] The self-reported global recovery question is reliable and valid in individuals with NAD secondary to a traffic collision [18, 20–22]. We defined recovery as feeling “all better (cured)” or “feeling quite a bit improved” [18, 20]. A participant was considered recovered on the first interview when recovery was reported.

#### Secondary outcomes: claim Closure

Claim closure has been used as an indicator of recovery in previous research [1, 23–25]. We defined claim closure as the number of days from the date of the injury to the date on which the claim was closed (i.e. payments ceased and a final agreement was reached between the insurer and the claimant) [25]. Closure usually coincides with the end of treatment, the attainment of maximal medical improvement, or with the end of income-replacement payments. Individuals with NAD who close their claims have lower neck pain intensity, better physical functioning and fewer depressive symptoms than those who keep their claims open [26, 27].

### Analysis

We used Cox regression to derive and replicate the prediction models [28]. We performed a complete case analysis. We described the association between predictors and time-to-recovery/time-to-claim-closure using hazard rate ratios (HRR) and 95% confidence intervals (CI). All analyses were conducted using SAS Version 9.3 and Stata MP 12. In the Saskatchewan cohort, we defined recovery as the time to first report of recovery with no subsequent report of having failed to recover. In the Ontario trial, we defined recovery as the time to first report of recovery. Therefore, recovery could occur at any follow-up period (6 weeks, 3, 6, 9 or 12 months) for self-reported recovery and at any time within the study period for claim closure. Observations on participants who did not recover were censored at the last report of non-recovery or at the 12-month follow-up. We handled ties using Efron approximation [28].

#### Univariate Associations

We examined the crude associations between each prognostic factor and the outcome using Cox regression. The statistical significance (p < 0.05) was assessed with the Wald Chi-square test [28]. Factors significantly associated with the outcome were included in the multivariable model. The proportionality assumptions were tested with Kaplan-Meier (categorical variables) curves and using time-interactions. Continuous measures (age, percent of body in pain, WDQ) were also categorized and tested as categorical variables.

### Multivariable analysis

#### Development of the Prediction Model

Prior to inclusion of potential prognostic factors in the multivariable models, we tested all variables to determine which were associated with outcomes of self-reported recovery and claim closure and we tested for multicollinearity. Pearson correlations were used to assess normally distributed variables and Spearman rank correlations to assess nominal and ordinal variables or variables with skewed distributions. Pairs of variables with correlations of r = 0.7 or more were considered as highly associated for inclusion in the same predictive model [28]. Second, we assessed the variance inflation factors (VIF) of each variable in each model. We considered VIF greater than 2.5 to designate multicollinearity [29].

We used three stages to develop our models. In Stage 1, we built a model that included Phase III prognostic factors available in the Saskatchewan dataset. Our datasets did not specifically ask about pain-related anxiety or worry; however, we made the assumption that participants answered about anxiety or worry related to their NAD pain-related symptoms. In Stage 2, we added prognostic factors supported by Phase I and II evidence. Finally, in Stage 3, we tested the contribution of additional clinically relevant variables. A lack of significant association was identified by HRR 95% confidence intervals that cross one [28]. We removed factors in a backward fashion, starting with the least statistically significant, until all associations remaining in the model were significant. We did not test for interactions.

We determined the predictive ability of each model using Harrell’s concordance C-statistic [3]. The C-statistic estimates the predictive ability of the model by computing the area under a receiver-operating curve (AUC) [3]. The C-statistic ranges from 0 to 1; a value of 0.5 or less denotes that the prediction is due to chance while values approaching 1 demonstrate better predictive ability [3].

#### Internal validation of the Prediction Model

We tested the internal validity of our predictive models using bootstrapping [3]. We performed 3000 repetitions per model. Our model was deemed internally valid if the standard error of the point estimate following bootstrapping did not change significantly compared to the error obtained at the development stage.

#### Repeating the Prediction Models in the Ontario Population

We tested our models by repeating them using data from a randomized controlled trial conducted in Ontario [3, 19]. We replicated the models developed in the Saskatchewan population by testing the Stage 1 and 2 models with the same or similar variables available in the Ontario population [Table 1]. In the Saskatchewan sample, we used a combination variable for disability that included “yes” responses for single questions of functional limitations related to work, study or other activities. In the Ontario dataset, disability was tested using the continuous baseline scores of the Whiplash Disability Questionnaire [30]. In the Saskatchewan sample, we used a percent body in pain variable developed from the body pain diagram and ranging from 0 to 100. In the Ontario dataset, body pain was represented by a 3-category variable stating whether the person had muscle, bone or joint pain. To test the impact of the body pain variable on the prediction of each model, we repeated the prediction models excluding this variable since its construct differed between the two populations.

**Table 1:** Descriptive Baseline Characteristics of the Samples from Saskatchewan and Ontario

	Province		
Baseline Prognostic Factors	Saskatchewan	Ontario	Phase of evidence*
	(N = 4917)	(N = 340)	
Age in years [mean (s.d.)]	38.3 (15.1)	40.5 (13.2)	I, II
Female [frequency (%)]	3263 (66.3%)	229 (67.3%)	I, II
Married or common law	2638 (53.6%)	184 (54.0%)	n/a
Employed (paid employment)	4138 (84.1%)	286 (84.1%)	I, II
Average Neck Pain [mean (s.d.)]	6.5 (2.1)^#^	5.7 (2.1)	I, II
Average Low Back Pain [mean (s.d.)]	3.8 (3.5)^##^	4.2 (3.3)	
CES-D scores [mean (s.d.)]	16.3 (12.0)^###^	15.4 (12.6)	n/a
Depression (> 27 points on CES-D) [frequency (%)])	984 (20.0%)	60 (17.7%)	I, II, III
Prior Neck Injury [frequency (%)]	1156 (23.6%)	2 (0.59)^+^	III
Post collision anxiety or worry [frequency (%)]	2149 (43.7%)	210 (62.0%)	I, II, III
Disability [frequency (%)]	3769 (76.6%)		I, II
Whiplash Disability Questionnaire [mean (s.d.)]		54.8 (29.3)	I, II
Concentration Problems [frequency (%)]	1221 (24.8%)	121 (35.6%)	n/a
Percent of Body in Pain [mean (s.d.)]	23.6 (15.7)	n/a^++^	n/a
Muscle, bone or joint problems^++^ [frequency (%)]			n/a
Doesn’t have it		197 (57.1)	
None or mild effect on life		118 (34.7)	
Moderate or severe effect on life		28 (8.2)	
Expectations of recovery [frequency (%)]	(5 missing values)		I, II, III
Get better soon	1224 (24.9%)	165 (48.5%)	
Get better slowly	2060 (41.9%)	101 (29.7%)	
Never get better	90 (1.8%)	3 (0.9%)	
Don’t know	1539 (31.3%)	71 (20.9%)	
Days since injury [median (range)]	9.0 (0–21)	6.0 (0–25)	none

s.d.=standard deviation; ^#^ 67 missing values; ^##^ 48 missing values; ^###^ 134 missing values;

*Obtained from the systematic review [2]

^+^ for Ontario, any previous injury claims for car accident;

^++^ for Ontario, the proxy variable for percent body in pain was the muscle, bone or joint problem

## Results

### Descriptive statistics

The Saskatchewan and Ontario samples included 4923 and 340 participants, respectively. Participants in both samples had similar baseline characteristics [Table 1]. Two thirds of participants were female (66. 3% in Saskatchewan; 66.7% in Ontario), most were employed (84.1% for both) and most presented shortly post-collision (9 days [range 0–21]; 6 days [range 0–25]). Participants had a mean age of 38.3 (s.d. 15.1) years in Saskatchewan and 40.5 (s.d. 13.2) years in Ontario. The mean baseline neck pain was 6.5 (s.d. 2.1) in Saskatchewan and 5.7 (s.d. 13.2) in Ontario and the 12-month follow-up rate (84.4%; 78.8%) was also similar. Finally, median time-to-recovery based on the global recovery question was similar (95 days in Saskatchewan and 98 days in Ontario). In the Saskatchewan (development) sample, 4810/4923 participants were analyzed in the final model. In the Ontario sample, 311/340 observations were analyzed in the final model.

### Univariable Associations

The proportionality assumption was met for all models. All potential prognostic factors identified in our systematic review were significantly associated with the outcomes.

#### Multivariable analysis

No multicollinearity was present in the development dataset. VIF values in all models ranged between 1 and 1.7.

### Time-to-recovery

#### Stage 1: prediction model with phase III prognostic factors

All prognostic factors with Phase III evidence were significantly associated with time-to-recovery and the predictive ability of the model based on the C-statistic was higher than chance [Table 2]. Predictors of recovery included in the model: prior neck injury claim with SGI; anxiety/worry (as proxies of pain-related or NAD-related anxiety and fear); depression (Centre for Epidemiological Studies Depression Scale: CES-D > 27) and expectation of recovery at baseline [Table 3].

**Table 2:** Harrel’s C-statistics by predictive model

Modeling Stage	Predictive Model	Phase of prognostic factor evidence	Time to recovery C-statistic (95% CI)	Time to claim closure C-statistic (95% CI)
Development	Stage 1	Phase III	0.626 (0.617, 0.636)	0.598 (0.588, 0.607)
	Stage 2	Phase I-III	0.644 (0.634, 0.653)	0.636 (0.626, 0.645)
Internal Validation (Bootstrapped models)	Stage 1	Phase III	0.626 (0.617, 0.636)	0.598 (0.588, 0.600)
	Stage 2	Phase I-III	0.644 (0.634, 0.653)	0.636 (0.626, 0.645)
External validation in Ontario	Stage 2	Phase I-III	0.654 (0.617, 0.692)	0.580 (0.539, 0.621)

CI = Confidence Interval; Stage 3 models did not significantly improve the predictive ability of Stage 1 and 2 models with the C-statistic ranging from 0.64–0.65 for both time to recovery and time to claim closure

**Table 3:** Clinical Prediction Models with Significant Prognostic Factors for Time to Self-reported Recovery

		Stage 1	Stage 2	
Baseline prognostic factor		HR (95% CI)	HR (95% CI)	
Age in years		-	0.993 (0.99, 0.99)	
Prior neck injury claim with SGI		0.68 (0.63, 0.74)	0.72 (0.66, 0.78)	
Post-collision Anxiety or Worry		0.89 (0.83, 0.95)	-	
Depression as > 27 on CES-D		0.85 (0.78, 0.93)	-	
Pain Drawing - Percentage		-	0.99 (0.99, 0.99)	
Neck pain intensity (NRS)		-	0.93 (0.92, 0.95)	
Headache intensity (NRS)		-	0.98 (0.97, 0.99)	
Disability		-	0.92 (0.85, 0.99)	
Expectations of recovery				
Never get better		0.25 (0.18, 0.35)	0.28 (0.20, 0.40)	
Get better slowly		0.66 (0.61, 0.72)	0.72 (0.67, 0.78)	
Don’t know		0.43 (0.40, 0.47)	0.49 (0.45, 0.54)	

* Abbreviations CI = confidence interval; SGI = Saskatchewan Government Insurance; HR = Hazards Rate

Stage 1 included prognostic factors obtained from published phase III studies

Stage 2 included prognostic factors obtained from published phase I and II studies

‡ Stage 3 models included clinically relevant variables not obtained from published evidence. Model composition varied and there are no final model betas to present

#### Stage 2: prediction models with phase I and II prognostic factors

The second stage model included age (in years), prior neck injury claim, percentage of body in pain, baseline neck pain and headache intensity, disability and expectation of recovery as statistically significant predictors of time-to-recovery [Table 3]. The C-statistic for this model was slightly superior to the previous model [Table 2].

#### Stage 3: prediction models with clinically relevant prognostic factors

The addition of clinically relevant variables did not improve the model built in Stage II (C-statistic ranged from 0.64 to 0.65).

### The final model

#### Time-to-recovery

Based on the Saskatchewan sample, the predictive ability of the Stage 2 model was higher than the Stage 1 model. Therefore, the final model demonstrated that older age, prior neck injury claim, greater percentage of body in pain, greater baseline neck pain, greater baseline headache intensity, disability and poor expectation of recovery are predictive of greater time-to-recovery [Tables 2 and 3].

## Time-to-claim-closure

Similarly in the Saskatchewan sample, we found that all prognostic factors with Phase III evidence remained significantly associated with time-to-claim-closure [Table 4], the predictive ability of the model based on the C-statistic was higher than chance [Table 2] and clinically relevant variables did not improve the model built in Stage II (C-statistic ranged from 0.64 to 0.65). Therefore, the final model predicting longer time-to-claim closure included: older age, prior neck injury claim, greater percentage of body in pain, greater intensity of baseline neck pain and headache intensity, disability, depression and poor expectation of recovery [Table 4].

**Table 4:** Clinical Prediction Models with Significant Prognostic Factors for Time to Claim Closure

		Stage 1	Stage 2
Baseline prognostic factor		HR (95% CI)	HR (95% CI)
Age in years		-	0.999 (0.99, 0.99)
Prior neck injury claim with SGI		0.71 (0.66, 0.77)	0.776 (0.72, 0.84)
Post-collision Anxiety or Worry		0.85 (0.80, 0.91)	-
Depression as > 27 on CES-D		0.82 (0.75, 0.90)	0.99 (0.99, 0.99)
Pain Drawing - Percentage		-	0.97 (0.96, 0.99)
Neck pain intensity (NRS)		-	0.98 (0.97, 0.99)
Headache intensity (NRS)		-	0.78 (0.72, 0.85)
Disability		-	0.91 (0.84, 1.00)
Expectations of recovery			
Never get better		0.36 (0.26, 0.50)	0.41 (0.30, 0.58)
Get better slowly		0.74 (0.68, 0.80)	0.82 (0.76, 0.89)
Don’t know		0.56 (0.51, 0.61)	0.64 (0.59, 0.71)

* Abbreviations CI = confidence interval; SGI = Saskatchewan Government Insurance; HR = Hazards Rate

Stage 1 model included prognostic factors obtained from published phase III studies

Stage 2 model included prognostic factors obtained from published phase I and II studies

‡ Stage 3 models included clinically relevant variables not obtained from published evidence. Model composition varied and there are no final model betas to present

### Internal validation of the Prediction Models

Bootstrapped models demonstrated similar predictive ability as the development models for time-to-recovery and for time-to-claim closure [Table 2].

### Repeating prediction models in the Ontario Population

The final models were repeated in the Ontario dataset [Table 2]. Baseline expectation category of ‘never get better’ was excluded from the repeated model due to the small sample size (n = 8); therefore, the validity results for that model are reported without values for that predictor response. Excluding two variables that differed in construct between study samples did not cause a significant change in the predictive ability of the models; with disability removed (Saskatchewan recovery model: C = 0.643, 95% CI 0.633–0.652; Ontario recovery model: C = 0.637, 95% CI 0.599–0.675) or percent of body in pain removed (Saskatchewan recovery model: C = 0.641, 95% CI 0.631–0.651; Ontario recovery model: C = 0.649, 95% CI 0.613–0.686).

## Discussion

### Summary of evidence

We developed and validated a predictive model of recovery using strong methodology and results from a best-evidence synthesis [2]. Our final model demonstrated that expectation of recovery, age, prior neck injury claim, percentage of body in pain, baseline neck pain and headache intensity, and disability are predictive of longer time-to-recovery. In addition, depressive symptoms were also predictive of longer time-to-claim closure. Models for both self-reported recovery and insurance claim closure included the same predictors with the exception of depression being an extra variable in the prediction of claim closure. However, these models had limited predictive ability.

### Comparison with previous literature

We identified twelve previous predictive models for the recovery of NAD secondary to traffic collisions. The previous studies used similar outcomes and follow-up periods. The most common outcomes reported in these studies were disability [4–7, 9, 10, 13], persistent neck pain [12, 14] and self-reported recovery [8, 11, 15]. Most studies assessed their outcomes at one-year post-injury [4–6, 9, 10, 12, 13]. Participants were recruited from various sources (e.g., emergency departments, general practitioner practices, rehabilitation clinics, insurance companies and advertisements). In addition, prognostic factors tested in the models and methods used to develop and validate the models varied greatly among studies.

Our results differ from previous predictive models due to methodological differences. Previous models were developed in small samples that limited the number of modelled prognostic factors. Small sample sizes can lead to model uncertainty and unreliable predictions [3]. We developed our models in a large, population-based inception cohort of patients with incident traffic-related NAD (N = 4923) and replicated them using data from a smaller randomized controlled trial with a similar patient population (N = 340). Important differences between our models and the ones proposed by Atherton et al.: (1) method of analysis (logistic regression); (2) ill-defined method of selection of prognostic factors; and (3) failure to report predictive ability statistics; (4) use of all incident injury cases [12]. Only one previous study internally validated their model [8] and five externally validated their model [5–7, 15, 16]. Three external validation studies included prognostic factors not available to us (i.e. measure of sympathetic vasoconstriction, range of motion assessments, hyperarousal subscale) [6, 7, 16]. One study did not use an appropriate sample to validate their model (floor effect) [5]. Finally, one study’s population may differ from ours since they recruited through emergency departments only [14].

### Clinical implications

We derived and validated a clinical prediction model for patients with post-collision neck pain. Our models predict self-reported global recovery and time to claim closure beyond chance; however, further investigation is needed before the models are recommended for clinical practice. Specifically, our models need to be validated in different populations and clinical settings, and an impact analysis needs to be conducted [31]. Impact analysis are necessary to determine the effect of a clinical prediction model on clinician behaviour and patient outcomes.

### Study strengths and limitations

Our study has strengths. We used a large, population-based inception-cohort to develop our models which gives robust model estimates and statistical certainty [3]. We replicated our models in Ontario, a distinct population with similar demographic and clinical characteristics [3]. Both cohorts included incident cases only. Finally, we used evidence-based methods to identify prognostic factors and applied data-based methods to identify additional clinically relevant prognostic factors.

Our study also has limitations. Although the Saskatchewan and Ontario samples had similar baseline characteristics, the methods of recruitment for the two studies were different. Our dataset provided a predetermined set of potential prognostic factors. Therefore, we were not able to test additional potential factors including passive coping, cervical radiculopathy, post-crash cold pain threshold, symptoms of Acute Stress Disorder/Post-Traumatic Stress Disorder and kinesiophobia. It is possible that adding these variables to our models could improve their predictive ability. Our anxiety or worry questions were not specifically pain-related and may have included generalized anxiety or worry. However, we assumed that participants answered about their NAD pain-related anxiety or worry since these questions were asked as part of a questionnaire about their NAD-related experience. Our models demonstrated a limited predictive ability; however, this limited predictive ability is predictive beyond chance. Considering that predictive ability of clinical judgement alone is unknown, a model that provides predictive capacity beyond chance adds some certainty to clinical practice. Therefore, we recommend the use of these predictive models until models with higher predictive ability are identified likely including predictive factors not included in our models. Expectation of recovery is the most predictive factor identified by our models and this is a factor that clinicians can focus on with their patients immediately to improve recovery.

Future prediction models should consider potential prognostic factors identified by the best-evidence synthesis that were not available in our datasets. Furthermore, future research should consider factors that have not been studied-to-date that could add to the limited predictive ability of current predictive models.

## Conclusions

We developed clinical prediction models that predict recovery and claim closure in individuals with NAD following traffic collisions. Prognostic factors included expectation of recovery, age, having a prior neck injury claim, percentage of body in pain, baseline neck pain and headache intensity, and disability. In addition, depressive symptoms remained predictive in the model predicting claim closure. Our models have limited predictive ability and require an impact analysis before being used in clinical settings.

### Electronic supplementary material

Below is the link to the electronic supplementary material.

Supplementary Material 1: TRIPOD Checklist

Supplementary Material 2: Additional variables tested in Stage 3 of the clinical prediction model development

## Data Availability

The datasets generated and/or analyzed during the current study are not publicly available due to lack of consent from participants.

## Abbreviations

AUC: area under a receiver-operating curve
CES-D: Centre for Epidemiological Studies Depression Scale
CI: 95% confidence intervals
HRR: Hazard rate ratios
NAD: neck pain and associated disorders
s.d.: Standard deviation
SGI: Saskatchewan Government Insurance
VIF: Variance inflation factors
WAD: Whiplash-associated disorders
WDQ: Whiplash Disability Questionnaire
