# Supplementary material for: Multivariable prediction models for the recovery of and claim closure related to post-collision neck pain and associated disorders
Source: Chiropr Man Therap. 2023 Aug 25;31:32. doi: 10.1186/s12998-023-00504-1 (PMC10464149; doi:10.1186/s12998-023-00504-1)
Supplement: Supplementary file 2 — Supplementary Material 2: Additional variables tested in Stage 3 of the clinical prediction model development [file 12998_2023_504_MOESM2_ESM.docx]

**Appendix A: Additional variables tested in Stage 3 clinical prediction model development**

| **Variable Domains** | **Inception Cohort Study**  **(Saskatchewan)** | **Whiplash Intervention Trial (Ontario)** |
| --- | --- | --- |
| Socio-demographic | sex, education, income, marital status, number of dependants, employment status | sex, education, income, marital status, number of dependants, employment status |
| Injury-related symptoms | Numbness, tingling or pain in arms or hands; numbness, tingling or pain in legs or feet; dizziness or unsteadiness; memory problems or forgetfulness; concentration or attention problems; vision problems; hearing problems; sleep problems; unusual fatigue or tiredness; sore jaw | Numbness, tingling, or pain in arm or hand; numbness, tingling, or pain in leg or foot; dizziness/unsteadiness; problems concentrating; vision problems; hearing problems; memory problems or forgetfulness; sleep problems; sore jaw; unusual fatigue or tiredness; |
| Pain intensity | Numerical Rating Scale for pain in:  1) low back; 2) arms; 3) hands; 4) legs; 5) face; 6) feet; 7) mid back pain | Numerical Rating Scale for pain in:  1) low back; 2) arms; 3) hands; 4) face; 5) legs; 6) feet; 7) mid back pain |
| Initial health care | Emergency room, physician, chiropractor, physiotherapist, massage therapist | Emergency room, physician, chiropractor, physiotherapist, massage therapist |
| Pre-injury comorbidities | Muscle or joint, allergies, diabetes,  high blood pressure, headache, cancer.  Problems with following system: breathing heart or circulation, digestive system, genito-urinary, neurological. | Muscle or joint, allergies, diabetes, high blood pressure, headache, cancer.  Problems with following system: breathing heart or circulation, digestive system, genito-urinary, neurological. |
